# Promising effects of xanthine oxidase inhibition by allopurinol on autonomic heart regulation estimated by heart rate variability (HRV) analysis in rats exposed to hypoxia and hyperoxia

**DOI:** 10.1371/journal.pone.0192781

**Published:** 2018-02-12

**Authors:** Stanisław Zajączkowski, Wiesław Ziółkowski, Piotr Badtke, Miłosz A. Zajączkowski, Damian J. Flis, Adam Figarski, Maria Smolińska-Bylańska, Tomasz H. Wierzba

**Affiliations:** 1 Department of Physiology, Medical University of Gdansk, Gdansk, Poland; 2 Department of Bioenergetics and Nutrition, Faculty of Rehabilitation and Kinesiology, Gdansk University of Physical Education and Sport, Gdansk, Poland; 3 Department of Clinical Anatomy, Medical University of Gdansk, Gdansk, Poland; 4 Department of Medical Chemistry, Medical University of Gdansk, Gdansk, Poland; University of Alabama at Birmingham, UNITED STATES

## Abstract

**Background:**

It has long been suggested that reactive oxygen species (ROS) play a role in oxygen sensing via peripheral chemoreceptors, which would imply their involvement in chemoreflex activation and autonomic regulation of heart rate. We hypothesize that antioxidant affect neurogenic cardiovascular regulation through activation of chemoreflex which results in increased control of sympathetic mechanism regulating heart rhythm. Activity of xanthine oxidase (XO), which is among the major endogenous sources of ROS in the rat has been shown to increase during hypoxia promote oxidative stress. However, the mechanism of how XO inhibition affects neurogenic regulation of heart rhythm is still unclear.

**Aim:**

The study aimed to evaluate effects of allopurinol-driven inhibition of XO on autonomic heart regulation in rats exposed to hypoxia followed by hyperoxia, using heart rate variability (HRV) analysis.

**Material and methods:**

16 conscious male Wistar rats (350 g): control-untreated (N = 8) and pretreated with Allopurinol-XO inhibitor (5 mg/kg, followed by 50 mg/kg), administered intraperitoneally (N = 8), were exposed to controlled hypobaric hypoxia (1h) in order to activate chemoreflex. The treatment was followed by 1h hyperoxia (chemoreflex suppression). Time-series of 1024 RR-intervals were extracted from 4kHz ECG recording for heart rate variability (HRV) analysis in order to calculate the following time-domain parameters: mean RR interval (RRi), SDNN (standard deviation of all normal NN intervals), rMSSD (square root of the mean of the squares of differences between adjacent NN intervals), frequency-domain parameters (FFT method): TSP (total spectral power) as well as low and high frequency band powers (LF and HF). At the end of experiment we used rat plasma to evaluate enzymatic activity of XO and markers of oxidative stress: protein carbonyl group and 8-isoprostane concentrations. Enzymatic activity of superoxide dismutase (SOD), catalase (CAT) and glutathione peroxidase (GPx) were measures in erythrocyte lysates.

**Results:**

Allopurinol reduced oxidative stress which was the result of hypoxia/hyperoxia, as shown by decreased 8-isoprostane plasma concentration. XO inhibition did not markedly influence HRV parameters in standard normoxia. However, during hypoxia, as well as hyperoxia, allopurinol administration resulted in a significant increase of autonomic control upon the heart as shown by increased SDNN and TSP, with an increased vagal contribution (increased rMSSD and HF), whereas sympathovagal indexes (LF/HF, SDNN/rMSSD) remained unchanged.

**Conclusions:**

Observed regulatory effects of XO inhibition did not confirm preliminary hypothesis which suggested that an antioxidant such as allopurinol might activate chemoreflex resulting in augmented sympathetic discharge to the heart. The HRV regulatory profile of XO inhibition observed during hypoxia as well as post-hypoxic hyperoxia corresponds to reported reduced risk of sudden cardiovascular events. Therefore our data provide a new argument for therapeutical use of allopurinol in hypoxic conditions.

## Introduction

Experimental and clinical data published to date have shown protective action of numerous antioxidants, which include antiatherogenic, antinflammatory and hypotensive effects. These molecules have also been shown to play a role in preventing endothelial dysfunction, vascular damage as well as protection from cardiac dysfunction caused by ischemia [1]. One such enzyme, xanthine oxidase (XO; EC 1.1.3.22), has been shown to play a key role in purine metabolism and is among the major endogenous sources of reactive oxygen species (ROS). Moreover, it is implicated in inflammatory processes and ischemic injury. XO catalyzes oxidation of hypoxanthine to xanthine and xanthine to uric acid, while O_2_ acting as a cofactor is reduced to reactive superoxide radical (O_2_^•−^). Following spontaneous dismutation the latter is converted to hydrogen peroxide (H_2_O_2_) which is a major final ROS product of XO action, especially in hypoxic or inflammatory conditions. Inhibition of XO activity with allopurinol has been shown to reduce oxidative stress and prevent oxidative stress-driven cellular damage as well as ischemic complications [2]. Allopurinol has been used to treat hyperuricemia and gout with relatively minor adverse effects. Beneficial effects of allopurinol, or its more stable metabolite oxypurinol, are evidenced in the case of vascular injury, inflammation [3], heart failure[4], ischemic heart disease [2,5], and also myocardial protection during cardiac or aortic surgery or post-ischemic reperfusion[6]. Experimental and clinical data indicated reduction of the risk of ventricular arrhythmias related to prolonged allopurinol use [7,8]. In contrast to favorable effects of various antioxidants observed in experimental studies and in a wide range of clinical settings, a number of randomized multi-center clinical trials performed over the last two decades, disclosed that chronic use of common commercially available antioxidants such as beta-carotene, vitamin A, vitamin E, vitamin C or selenium has been associated with critical cardiovascular events, sudden death and an increase of all-cause mortality [9–11]. Intriguing point is that neither data from structural nor biochemical/biophysical studies have explained the nature of these adverse effects. ROS, and in particular O_2_^•-^, were shown to contribute in the arterial oxygen pressure sensing—thereby being involved in autonomic heart regulation. Systemic hypoxia apparently activates carotid bodies (CB), located in the carotid artery bifurcation, to trigger complex cardiorespiratory response, whereas hyperoxia inactivates the oxygen-sensing cells [12,13]. Although detailed mechanism of chemoreflex activation is still a matter of controversy, the evidence seems to indicate a pivotal role for inhibition of outward TASK K^+^ channel in CB, which results in an increased frequency of neural discharge from CB to the cardiorespiratory centers in the brainstem [14,15]. ROS such as O_2_^•-^ or peroxides generated in the CB, do not only affect the redox state of the sensing cells but they can also oxidize TASK K^+^ channels, thus regulate their conductance. It is generally accepted that the chemoreflex activation depends on availability of oxygen and the redox state of the sensing cells. The literature suggests that increased local oxygen availability is proportional to enhanced ROS production and oxidative stress, whereas low O_2_ concentrations trigger the response to hypoxia as a result of compromised ROS generation. Suppression of endogenous oxygen radicals by antioxidants was hypothesized to induce the reflex cardiovascular response similar to that evoked by systemic hypoxia, with an increase of sympathetic drive of autonomic regulation of heart rhythm and ensuing increased risk of severe cardiac events [16–18]. Older experimental data which referred to the regulatory effects of antioxidants seemed to be inconsistent. Histidine, a powerful scavenger of singlet oxygen molecule (^1^O_2_), or trolox, a water soluble analogue of vitamin E, elicited an increase of sympathetic discharge to the rat heart [19,20], whereas ascorbic acid enhanced vagal control over the rat heart at doses up to 10 mg/kg, but at higher doses it increased sympathetic activity [21]. On the other hand, prolonged supplementation with synthetic nitroxide antioxidant, tempol (4-hydroxy-2,2,6,6-tetramethylpiperidin-1-oxyl), increased autonomic control in hypoxia as well as hyperoxia in hypertensive rats (SHR, or nitric-oxide deficient) [22].

Notwithstanding ROS suppression by allopurinol and its wide range of antioxidant effects in a large-scale clinical trial, OPT-CHF study did not show any clinical benefit of allopurinol use in symptomatic patients with congestive heart failure and related oxidative stress [23]. Therefore, despite suggested role of antioxidants in the chemoreceptor-dependent cardiovascular regulation, the regulatory effect of XO inhibition has not been yet elucidated. However, analysis of beat-to-beat changes in the heart period known also as heart rate variability (HRV) analysis seems to offer a valid non-invasive insight into the autonomic heart control [24]. Physiological sinus rhythm is variable and is characterized by complex oscillations which originate from neurogenic, respiratory and humoral components [25,26]. Decomposition of the RR-interval time-series derived from the sinus rhythm ECG with use of linear or non-linear methods of the HRV analysis allows for discrimination of the vagal and sympathetic discharge. Periodic fluctuations of vagal activity are of much higher frequency than the sympathetic discharge. Unlike sympathetic reflex axes between the heart and integrative regulatory neurons located in brainstem, the vagally driven feedbacks operate fast, so that the regulatory response may be completed within the given heart cycle. Although the physiological meaning of HRV parameters have not been fully understood [27], it is generally accepted that the traditionally used approach of HRV analysis in time-domain or frequency-domain, provide reliable measures of vagal tone and sympathetic discharge [24,27]. The standard deviation of all RR-intervals in a beat-to-beat time series (SDNN) is a major time-domain indicator of the overall autonomic regulatory control whereas total spectral power (TSP) is a corresponding frequency-domain measure. Root mean square of successive RR interval (RRi) differences (rMSSD) or its frequency-domain equivalent high frequency spectral power (HF) represent high frequency oscillations of RRi, and therefore estimates vagal activity [28]. The ratio of LF to HF spectral power (LF/HF) and its recently recommended time-domain surrogate, SDNN/rMSSD, reflect relative contribution of sympathetic drive in autonomic control [24,29].

In this study, performed on unrestrained rats supplemented with allopurinol, we aimed to evaluate the effect of XO inhibition on HRV in standard normoxic conditions and during activation or inhibition of the peripheral chemoreflex by controlled hypobaric hypoxia as well as normobaric hyperoxia.

## Materials and methods

This study was carried out in strict accordance with the recommendations in the Guide for the Care and Use of Laboratory Animals of the National Institutes of Health. The protocol was approved by the Local Ethics Committee For Animal Experiments of the Medical University of Gdansk (consent no. 07/2011). Experiments were performed with all efforts made to minimize animal suffering. Sixteen male Wistar rats (350g) obtained from Medical University of Gdansk Breeding Laboratory (Gdansk, Poland) were used in this study. The animals were fed and watered *ad libitum* and kept under 12:12 hours light-dark cycle in standard atmospheric conditions. In the week preceding the proper procedure the rats were regularly habituated to the experimental environment. Then, the rats were anesthetized intraperitoneally with sodium pentobarbital (50mg/kg; Sigma-Aldrich Chemie GmbH, Munich, Germany) in accordance with institutional guidelines. Three silver ECG electrodes were implanted subcutaneously and exteriorized on occipital area. Following 48 hour recovery the experimental protocol was performed on conscious and unrestrained animals.

### Experimental protocol

The rats were placed in a transparent polycarbonate chambers (Thermo Scientific Polycarbonate Desiccator; Nalgene, USA) with enough room for free movements. To provide controlled conditions of hypobaric hypoxia or hyperoxia the chambers were connected to vacuum pump (KNF Pump Laboport N811KN.18; KNF Neuberger GmbH, Freiburg, Germany) or oxygen tank through the automatic adjustable pressure regulator (Vacuum regulator VAR; ROTH, Karlsruhe, Germany). Pressure inside the chambers were monitored with pressure sensor provided by PowerLab 26T (ADInstruments, Sydney, Australia). To provide stable CO_2_ tension during all phases of the experiment chambers were connected with external environment through 25-cm-long PE-10 tubing of high-resistance to air-flow (Clay-Adams, Parsippany, USA). All rats underwent the same experimental procedure in three consecutive days. The daily procedure took four hours and consisted of four subsequent phases: normobaric normoxia (i.e. baseline conditions), controlled hypobaric hypoxia applied for chemoreflex activation and for evoking of oxidative stress, controlled normobaric hyperoxia to suppress chemoreflex response, and the final recovery phase in standard normoxic conditions. Initially the rats remained under standard atmospheric conditions in well ventilated chambers (1-h normobaric normoxia). Then, during controlled hypobaric hypoxia (1h); pressure within the chambers were slowly reduced by 400mmHg within the initial 10 min. In the 3-rd phase, normobaric hyperoxia (1h), the chambers were filled with oxygen to provide at least 90% oxygen concentration. Finally, the chamber was ventilated and the rats were maintained in normobaric normoxic conditions for 1 h (recovery period). The animals were randomly divided into two groups: control (saline, N = 8) and allopurinol (N = 8). On first day of the experiment, the animals from the allopurinol group were injected with vehicle (1mg/kg ip.) containing dimethyl sulfoxide (DMSO, Sigma, St. Louis, USA; solvent for allopurinol) before being placed in the experimental chambers. Following the 4-h chamber procedure the rats were injected with XO inhibitor allopurinol (Sigma-Aldrich Chemie GmbH, Munich, Germany): 5mg/kg ip. The following day the dose was repeated prior to the procedure. Since literature data referred to the dose of allopurinol that would effectively inhibit XO in the rat were inconsistent, we decided to test two doses: low– 5 mg/kg, and high– 50 mg/kg [30–32]. The higher allopurinol dose was injected twice: after completion of the 2-nd day procedure and prior to the 3-rd day experiment. The control group received isotonic saline instead. According to the previous reports the applied pattern of allopurinol administration should provide effective reduction of XO activity in rats during the experimental sessions [33]. Allopurinol was freshly reconstituted in DMSO prior to each administration.

### Chemical analysis

At the end of experimental procedures rats were sacrificed by decapitation for sample collection. Blood was collected into heparinized vials and centrifuged at 1000 × g for 10 minutes at 4°C. The buffy coat was discarded. Plasma was collected to Eppendorf vials and stored at -80°C for further analysis. The packed erythrocytes were added to 4-fold excess of ice-cold HPLC-grade water. The obtained lysate was centrifuged at 10000 × g for 15 minutes at 4°C. Supernatant was collected to separate Eppendorf vials and also stored at -80°C, as indicated above.

#### Plasma protein oxidation assay

Protein carbonylation, which is a marker of protein oxidation was determined according to method of Oliver et al.[34]. Each plasma sample (100μl) was mixed with 100μl of 20mM 2,4-dinitrophenylhydrazine–DNPH solution (Sigma-Aldrich Chemie GmbH, Munich, Germany), whereas the respective control sample consisted of plasma and 100μl 2M HCl (Sigma-Aldrich Chemie GmbH, Munich, Germany). The samples were incubated at room temperature for 60 min, shaking continuously. The proteins were precipitated by an addition of 500 μl of 20% trichloroacetic acid (Sigma-Aldrich Chemie GmbH, Munich, Germany) and centrifuged at 1000 × g for 7 min. The pellet was washed three times with 1 ml of 1:1 (v/v) ethanol/ethyl acetate (POCh S.A., Poland; Sigma-Aldrich Chemie GmbH, Munich, Germany) with centrifugation as previously. After the final wash the pellet was drained off a visible liquid and left to dry completely, then it was reconstituted with 1 ml of 10mM sodium phosphate buffer, pH 6.5 containing 6M guanidine (Sigma-Aldrich Chemie GmbH, Munich, Germany). Then the mixture was incubated at 50°C with continuous shaking until the pellet dissolved. The absorbance was measured at the wavelength of 280 nm and 360 nm for protein and carbonyl content respectively (ND-1000 UV-Vis Spectrophotometer,Thermo Scientific, USA).

#### Plasma lipid peroxidation assay

Isoprostanes 8-iso-PGF2α plasma concentration was measured using a commercially available kit (Cayman Chemicals, Ann Arbor, USA) according to the manufacturer instruction. The assay is based on the competition between 8-isoprostane and an 8-isoprostane-acetylcholinesterase conjugate for 8-isoprostane-specific rabbit antiserum binding sites. The assay use the rabbit antiserum-8-isoprostane complex which binds to the rabbit IgG mouse monoclonal antibody. After Ellman’s reagent was added, solution turned yellow and was measured spectrophotometrically at 412 nm (Microplate reader model 680XR, Bio-Rad Laboratories, Inc, Hercules, USA).

#### Antioxidant enzyme activity assay

XO, superoxide dismutase (SOD; EC 1.15.1.1) and glutathione peroxidase (GPx; EC 1.11.1.19) activities were assessed using dedicated Cayman Assay Kits (Cayman Chemicals, Ann Arbor, USA) according to the manufacturer’s detailed instruction. Catalase (CAT; EC 1.11.1.6) activity was measured by using method described by Aebi [35]. SOD, CAT and GPx activity were measured in erythrocyte lysate, whereas XO activity was assessed in blood plasma. The XO assay is based on a multistep enzymatic reaction during which XO causes the formation of highly fluorescent chemical compound resorufin. Resorufin fluorescence was analyzed with an excitation wavelength of 520–550 nm and an emission wavelength 585–595 nm (GloMax-Multi+, Promega Corporation;Madison, USA). To detect superoxide radical produced by XO the SOD assay uses a tetrazolium salt. During reaction tetrazolium salt was reduced into formazan dye by XO. Absorbance was read at 440–460 nm. GPx activity was measured indirectly by coupled reaction with glutathione reductase (GR). Oxidized glutathione from GPx reaction was reduced by GR and NADPH. The decrease in absorbance at 340 nm follows an oxidation of NADPH to NADP^+^. To measure CAT activity a mixture of H_2_O_2_, in phosphate buffer, pH 7.0, and necessary volume of sample was prepared. The molar requisite coefficient of 43.6 M cm^-1^ was used. The activity was calculated by measuring the decrease in absorbance at 240 nm (Super Aquarius CE9200 spectrophotometer; Cecil Instruments Ltd., Cambridge, UK).

### Heart rate variability (HRV) analysis

High resolution (4kHz) electrocardiogram (ECG) was continuously recorded with use of PowerLab 26T (AdInstruments, Sydney, Australia). All QRS complexes were thoroughly checked to avoid false positive detections and missed beats. RR intervals (RRi) were identified using automatic R-peak detection of ECG (LabChart 7 Pro software, AdInstruments, Sydney, Australia; Microsoft Excel 2013, USA). The sinus rhythm time-series of 1024 consecutive RRi were obtained between the 35^th^ and 55^th^ minute of ECG recording in each experimental phase when rats shown mostly unconstrained behavior. RRi, which differ by more than 3 SDs (standard deviation) from the previous RR interval or sporadic artifactual peaks, were automatically corrected by dedicated software (Kubios Pro 2.0, Kuopio, Finland). The smooth priors method with λ = 2000 was used for smoothing data set prior to spectral analysis of heart rate variability (HRV). After correction of RRi-time-series to obtain normal-to-normal RR intervals (NN-time-series), HRV analysis was performed with use of KubiosPro2.0 software (Kuopio, Finland) and Microsoft Excel (Microsoft, 2013, USA). HR (heart rate), mean RRi, SDNN (the standard deviation of all normal NN intervals) and rMSSD (square root of the mean of the squares of differences between adjacent NN intervals) were taken as a representative time-domain parameters. The frequency-domain (spectral) parameters were assessed with fast Fourier transform (FFT) algorithm with estimation of spectral density using Welch’s periodogram. Spectra were assessed from the entire selected 1024-NN window without data overlapping [36]. Predefined spectral bands adjusted to the rat were set at 0.2–0.75 Hz (low frequency, LF) and 0.75–2.5 Hz (high frequency, HF) and were expressed in absolute values (ms^2^) [37].

### Statistical analysis

Statistical analysis was performed by the Statistica 12 (StatSoft, Tulsa, USA) and GraphPad Prism 5 (GraphPad Software, La Jolla, California, USA) softwares. All data sets were tested for normality with Shapiro-Wilk test. Statistical analysis was based on Mann-Whitney, Wilcoxon test and Student t-tests, depending whether data have normal or non-normal distribution. The Pearson linear regression method was applied to calculate the relation between enzymatic activity and markers of oxidative stress. P values <0.05 were considered statistically significant. All data are shown as mean values ± standard error of the mean (±SEM).

## Results

### Inhibition of XO by allopurinol

**Fig 1:**
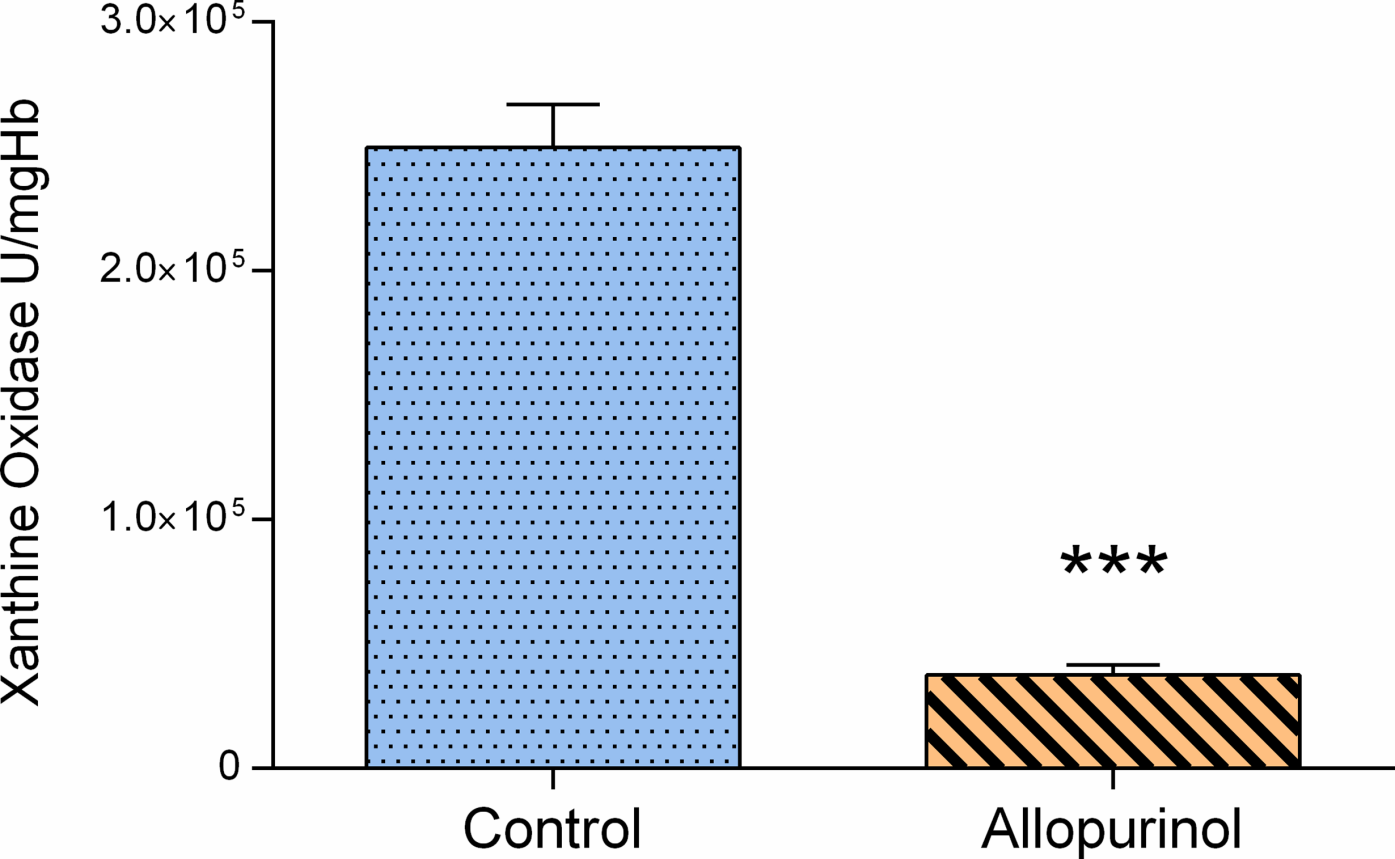
XO activity in plasma per 1 mg of hemoglobin (μU/mgHb). Blue bar indicates control group (N = 8), orange bar–rats pretreated with allopurinol (50 mg/kg ip.). Data shown as mean ± SEM *** p<0.001, allopurinol vs. control group according to Mann-Whitney test.

In rats injected with allopurinol XO activity was reduced by 87% compared to their baseline reference as shown on Fig 1.

### Effects of allopurinol on antioxidant enzyme activity

**Fig 2:**
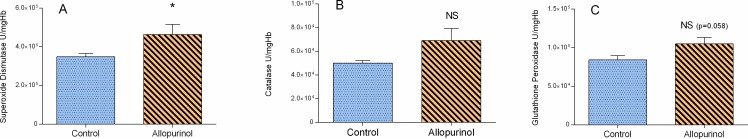
**Effect of XO inhibition by allopurinol (50mg/kg) on activity of antioxidant enzymes in erythrocyte lysate: superoxide dismutase (A), catalase (B), glutathione peroxidase (C).** Blue bar indicates control group, orange bar–rats treated with allopurinol. Data shown as mean ± SEM * p<0.05, NS–non-significant, allopurinol vs. control group (N = 8; Fig A: Mann-Whitney test; Fig B and C: t-test).

Allopurinol (50 mg/kg ip.) resulted in an increased activity of all tested antioxidant enzymes. Although an increase of SOD activity was the only significant change (p = 0.038; Fig 2A), a non-significant trend of increased CAT or GPx activity was not negligible (p = 0.117; Fig 2B; and p = 0.058, Fig 2C, respectively).

### Markers of oxidative stress

**Fig 3:**
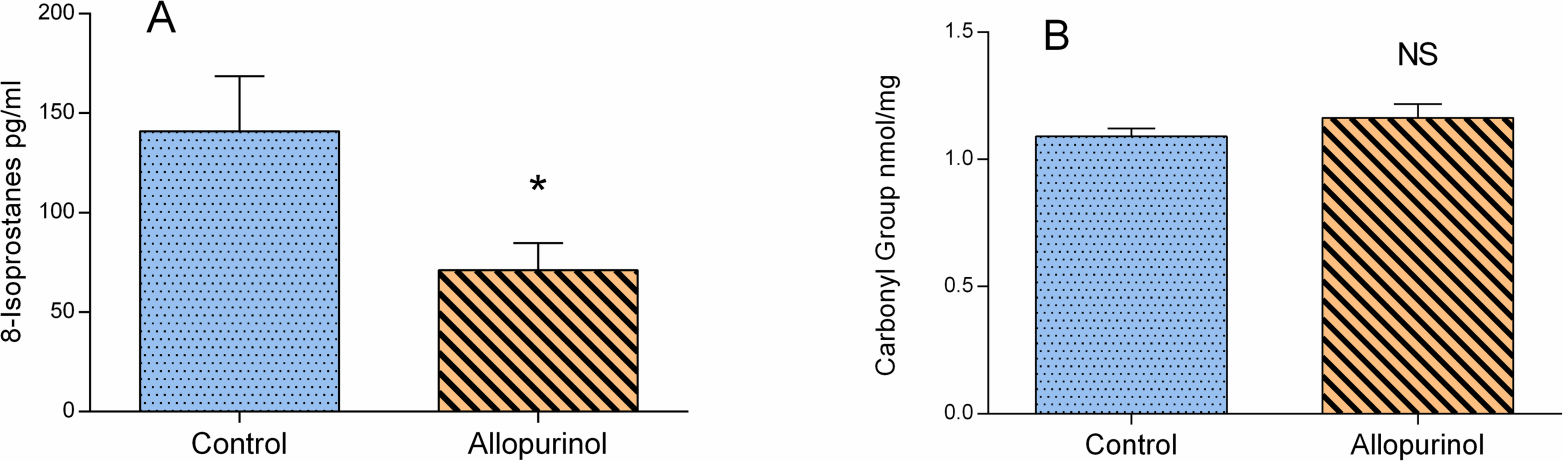
**Effect of XO inhibition by allopurinol on markers of oxidative stress in plasma: 8-isoprostanes (A) and protein carbonyl group (B).** Blue bar indicates control group, orange bar–rats treated with allopurinol. Data shown as Mean ± SEM according to Student t-test, * p<0.05, NS–not significant, allopurinol vs. control group.

Following allopurinol supplementation plasma concentration of 8-isoprostanes was significantly decreased (Fig 3A) indicating protection against lipid peroxidation. XO activity was closely related to plasma lipid peroxidation as found by significant reciprocal relationship between plasma concentration of plasma 8-isoprostanes and XO activity (r = -0.7279, p = 0.041). XO inhibition did not result in a relevant change of plasma protein carbonyl groups (Fig 3B). Consistently, protein carbonyl group concentration and XO activity measured at the end of the experimental protocol were not closely related as shown by lack of significant correlation (r = 0.1791, NS).

### Effect of hypoxia or hyperoxia on HRV in rats with baseline XO activity

As shown in Table 1 DMSO, used as a solvent of allopurinol, did not influence HRV as shown by comparison of HRV parameters in control group initially preinjected with vehicle and the rats from allopurinol group pretreated with DMSO (1 ml/kg) before allopurinol administration [38]. At the beginning of the experimental procedure, i.e. before allopurinol or vehicle administration, the HRV responses to hypoxia, hyperoxia or the recovery normoxia were comparable in the control and allopurinol groups (Table 1).

**Table 1:** HRV in the control and allopurinol group and allopurinol groups before allopurinol administration.

	Control group				Allopurinol group: before allopurinol administration			
	normoxia	hypoxia	hyperoxia	recovery	normoxia	hypoxia	hyperoxia	recovery
**RRi**	190±7.72	206±10.12	222±4.76*	209±4.25*	181±5.58	211±8.35*	217±7.41**	203±9.90*
**SDNN**	4.03±0.35	4.41±0.80	3.16±0.37*	4.98±0.60	4.23±0.69	6.08±1.12	2.72±0.40*	4.11±0.60
**rMSSD**	3.53±0.35	4.14±0.74	2.84±0.43	4.63±0.80	3.29±0.45	5.39±0.59*	2.58±0.31	3.73±0.59
**SDNN/rMSSD**	1.17±0.09	1.09±0.09	1.17±0.13	1.18±0.14	1.26±0.05	1.10±0.13	1.04±0.04*	1.13±0.06
**TSP**	16.15±2.11	19.44±6.64	7.81±1.23**	20.67±3.51	19.72±7.20	29.94±7.02	6.90±2.31	14.42±3.95
**LF**	2.43±0.13	2.55±0.98	1.25±0.35*	4.06±0.90	4.18±1.63	5.75±1.84	1.68±0.68	4.65±1.15
**HF**	3.73±0.74	4.16±1.24	2.32±0.64	6.53±1.76	3.52±1.20	6.37±1.09*	1.66±0.31	5.04±1.48
**LF/HF**	0.84±0.18	0.64±0.14	0.81±0.37	0.87±0.25	1.17±0.13	1.03±0.43	0.90±0.23	1.18±0.30

All differences between the control and the allopurinol group at a given phase of the experiment (normoxia, hypoxia, hyperoxia, recovery) were insignificant (Student t-test or Mann-Whitney test). Data shown as mean ± SEM.

* p<0.05 ** p<0.01 indicate differences within each group (control, allopurinol) in comparison to respective normoxia (Student t-test or Wilcoxon test).

RRi—RR interval, SDNN—the standard deviation of all normal NN intervals, rMSSD—square root of the mean of the squares of differences between adjacent NN intervals, TSP—total spectral power, LF—low frequency spectral power, HF—high frequency spectral power.

### HRV responses to hypoxia or hyperoxia

**Fig 4:**
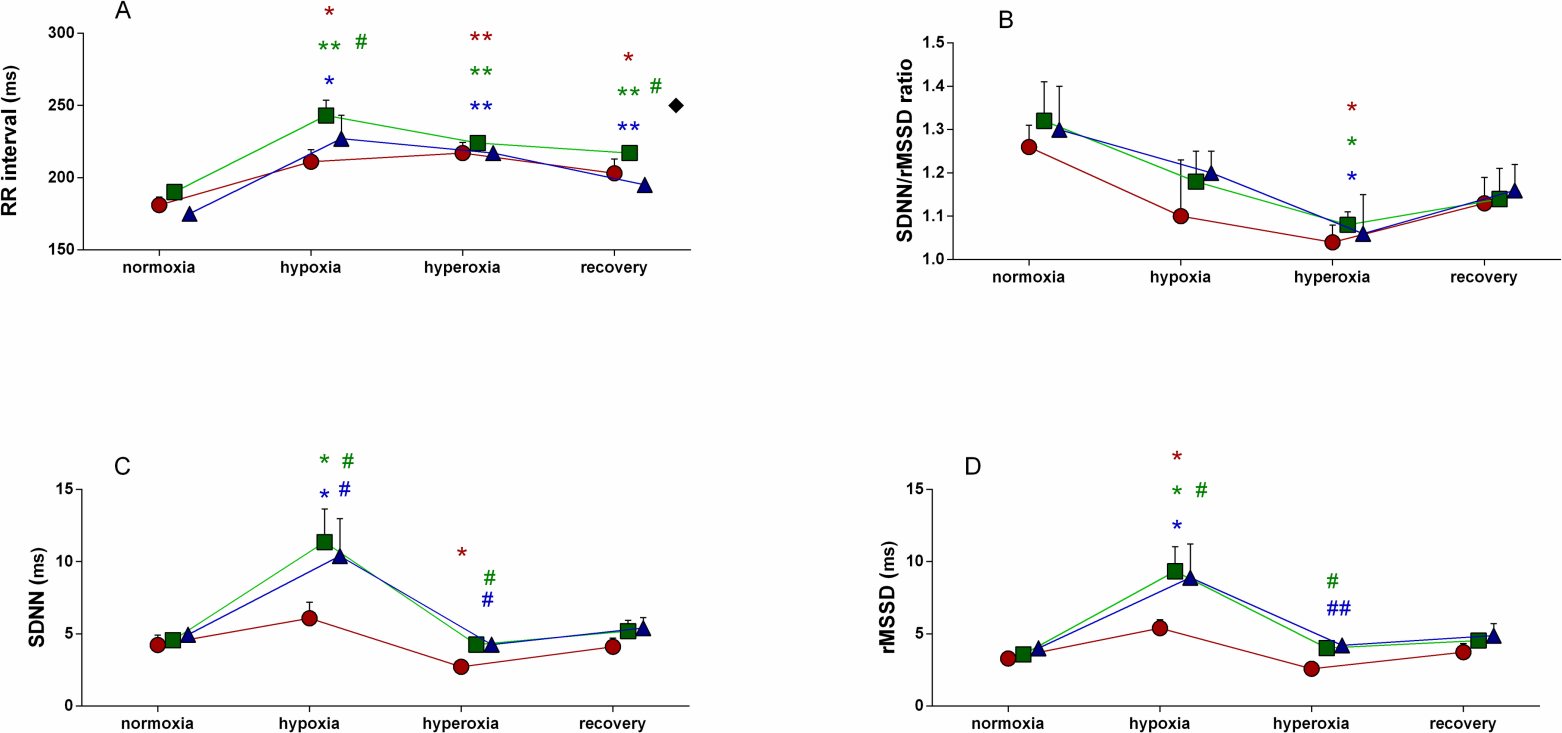
Effects of hypoxia and hyperoxia on time-domain HRV parameters. Data shown as mean ± SEM. Only significant values are tagged. Red color indicates allopurinol group before allopurinol administration (AGB), green—allopurinol group after injection of 5mg/kg of allopurinol, blue—allopurinol group after injection of 50mg/kg of allopurinol. * p<0.05 hypoxia, hyperoxia or recovery vs. normoxia, ** p<0.01 hypoxia, hyperoxia or recovery vs. normoxia in each group; green **#** p<0.05 – 5mg/kg of allopurinol vs. AGB at the same condition, green **##** p<0.01 – 5mg/kg of allopurinol vs. AGB at the same condition; blue **#** p<0.05 – 50mg/kg of allopurinol vs. AGB at the same condition, blue **##** p<0.01 – 50mg/kg of allopurinol vs. AGB at the same condition. ♦ p<0.05 – 5mg/kg vs. 50mg/kg of allopurinol.

**Fig 5:**
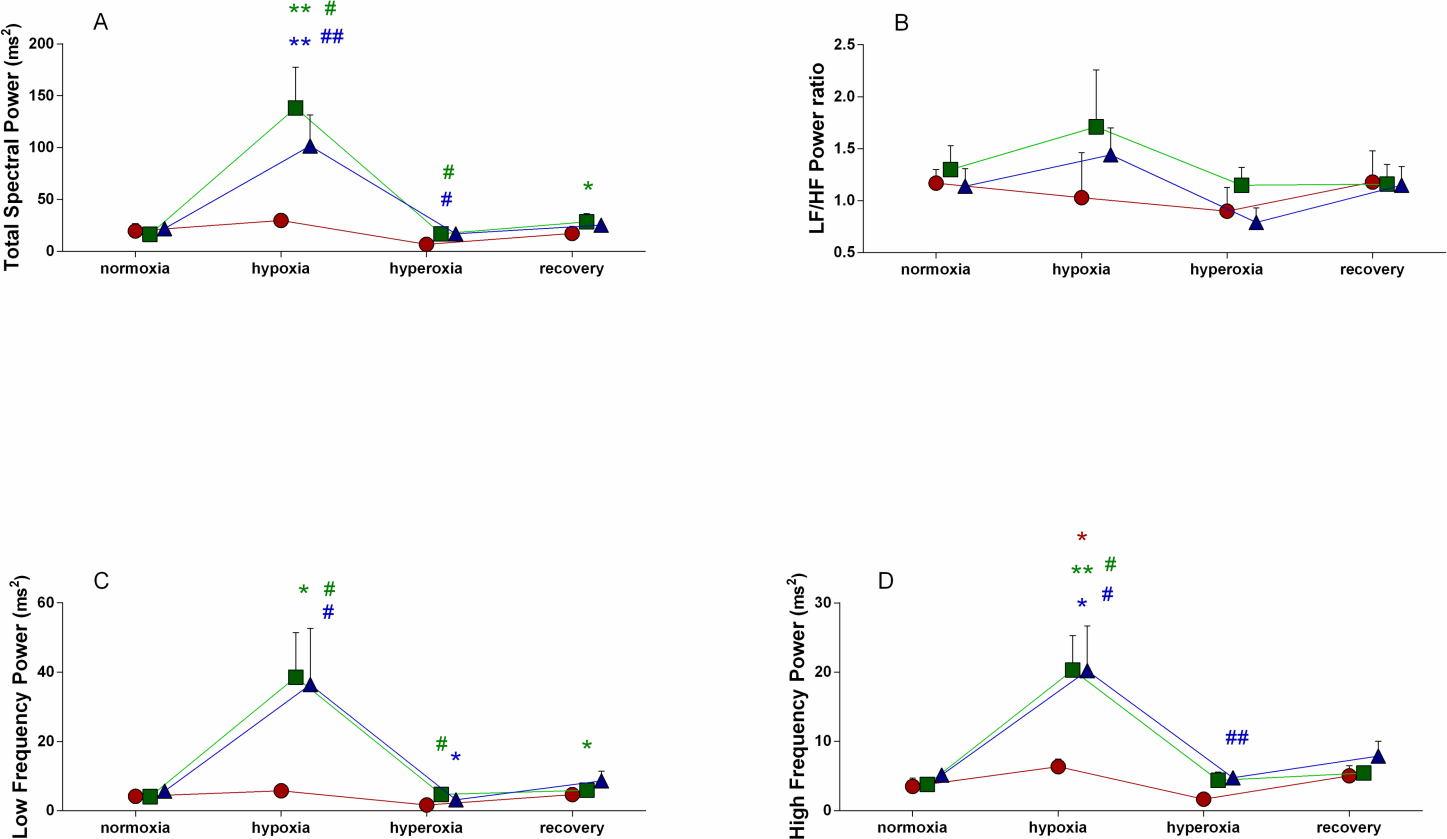
Effects of hypoxia and hyperoxia on frequency-domain HRV parameters. Data shown as mean ± SEM. Only significant values are tagged. Red color indicates allopurinol group before allopurinol administration (AGB), green—allopurinol group after injection of 5mg/kg of allopurinol, blue—allopurinol group after injection of 50mg/kg of allopurinol. * p<0.05 hypoxia, hyperoxia or recovery vs. normoxia, ** p<0.01 hypoxia, hyperoxia or recovery vs. normoxia in each group; green **#** p<0.05 – 5mg/kg of allopurinol vs. AGB at the same condition, green **##** p<0.01 – 5mg/kg of allopurinol vs. AGB at the same condition; blue **#** p<0.05 – 50mg/kg of allopurinol vs. AGB at the same condition, blue **##** p<0.01 – 50mg/kg of allopurinol vs. AGB at the same condition.

We have observed that both hypoxia and hyperoxia influenced HRV (Figs 4 and 5). Since HRV parameters assessed in the first day of procedure, i.e before allopurinol administration were consistent in the both tested groups we analyzed effects of hypoxia or hyperoxia within the one combined group (N = 16, Table 2). Data from each group are also shown in Table 1. Hypoxia resulted in a significant reduction of HR from 326±7 beats/min (BPM; range 273÷367 to 291±8; range 231÷349 BPM; p = 0.0004), that remained at the lower level in hyperoxia (275±5; range 241÷331 BPM; p = 0.0001). An incomplete recovery trend was observed during subsequent 60-min of normoxic recovery (294±8; range 261÷385 BPM; p = 0.003). Hypoxic conditions did not alter overall HRV as shown by unchanged time- and frequency-domain HRV parameters: SDNN, TSP and LF. Interestingly, hypoxia enhanced vagal discharge as shown by significant increase of rMSSD (p = 0.02), and a nonsignificant increasing tendency of HF (p = 0.08) In turn, in the following hyperoxia both rMSSD and HF were reduced below the baseline hypoxic level (p = 0.03 and p = 0.02, respectively). During either hypoxia or hyperoxia only minor, modest decrease of the time-domain or spectral indexes of the sympathovagal balance: LF/HF or SDNN/rMSSD were observed (p ranging from 0.07 to 0.18).

**Table 2:** Effect of hypoxia or hyperoxia on HRV in baseline conditions before allopurinol administration.

	Combined group			
	normoxia	hypoxia	hyperoxia	recovery
**RRi**	185±4.45	209±5.96 ***	219±4.04 ***	206±4.92 **
**SDNN**	4.13±0.35	5.24±0.66	2.94±0.25 **	4.55±0.4
**rMSSD**	3.41±0.26	4.76±0.46 *	2.71±0.24*	4.18±0.46
**SDNN/rMSSD**	1.22±0.04	1.09±0.07	1.11±0.06	1.16±0.07
**TSP**	17.94±3.42	24.69±4.57	7.35±1.19 **	19.04±2.42
**LF**	3.30±0.77	4.15±1.03	1.46±0.35 **	4.36±0.66
**HF**	3.62±0.64	5.27±0.80	1.99±0.33 *	5.79±1.05
**LF/HF**	1.01±0.11	0.84±0.21	0.86±0.20	1.03±0.18

Data shown as mean ± SEM. Only significant values are tagged (Student t-test or Wilcoxon test)

* p<0.05 ** p<0.01 *** p<0.001 indicate differences in comparison to normoxia.

RRi—RR interval, SDNN—the standard deviation of all normal NN intervals, rMSSD—square root of the mean of the squares of differences between adjacent NN intervals, TSP—total spectral power, LF—low frequency spectral power, HF—high frequency spectral power.

### Regulatory effects of allopurinol effect

#### Low dose

Allopurinol (5 mg/kg) driven inhibition of XO caused further decrease in HR during hypoxia (from 288±11; range 257÷349 to 252±10 BPM; range 211÷294 BPM; p = 0.017) in comparison to hypoxia before allopurinol administration. Allopurinol enhanced overall autonomic activity in hypoxic conditions which was manifested by an increase of SDNN from 6.08±2.97 to 11.36±6.07ms (p = 0.04; Fig 4C), TSP from 29.94±18.58 to 138.34±103.94ms^2^ (p = 0.02; Fig 5A) and LF from 5.75±4.88 to 38.56±34.09ms^2^ (p = 0.02; Fig 5C). Under hypoxic as well as hyperoxic conditions parasympathetic drive was also strengthened as indicated by increased rMSSD p = 0.04 and p = 0.03, respectively: Fig 4D) and HF p = 0.04, and p = 0.04, respectively; Fig 5D). Indexes of autonomic balance: LH/HF (Fig 5B) and SDNN/rMSSD (Fig 4B) remained unchanged.

#### High dose

Compared the HRV variables estimated after supplementation with the low allopurinol dose (5 mg/kg), the subsequent administration of the higher dose (50 mg/kg) did not evoke any substantial changes. The overall autonomic activity: TSP, LF, SDNN (Figs 4A and 4C and 5C) as well as the vagal drive: rMSSD and HF (Figs 4D and 5D) remained elevated compared to the reference conditions before allopurinol administration.

## Discussion

The major finding of this study, is that inhibition of XO significantly influenced neurogenic regulation of the heart rhythm during controlled hypobaric hypoxia and following hyperoxia. Allopurinol supplementation resulted in a significantly increased overall autonomic control including an increased vagal drive in the hypoxic conditions and also in the subsequent hyperoxia. Such profile of the regulatory response is most likely beneficial since dominant neurogenic control upon the intrinsic sinoatrial node pacemaker with prevalent vagal discharge is prerequisite for stable heart rhythm and is protective against ventricular arrhythmias [26,39,40].

HRV analysis has been widely used in human studies and clinical practice for more than two decades [24] for noninvasive evaluation of the neurogenic regulation of the heart, but only a few studies was focused on the regulatory effects of antioxidants. The rat HRV spectra resembles those derived from humans, showing two principal frequency components: the LF and HF [41,42]. In our study HRV analysis performed in time-domain and frequency-domain provided consistent data: direction and range of HRV changes induced by XO inhibition or hypoxic/hyperoxic challenge were equivalent. As shown in Figs 4C and 5A, XO inhibition resulted in an increase of SDNN and TSP in hypoxia but in hyperoxia we observed the decrease of these parameters. Although both hypoxia and the post-hypoxic hyperoxia were reported to induce oxidative stress [43,44], the different direction of the HRV changes observed in hypoxia or hyperoxia is consistent with the hypoxic chemoreflex activation followed by its hyperoxic inhibition [17,45].

Lack of significant differences between the effects of the two tested doses, 5 and 50 mg/kg, may suggest that nearly maximal modulation of the autonomic discharge was elicited by the smaller dose. TSP or SDNN reflect overall fluctuations of RRi-time-series in selected period. High TSP or SDNN suggests that heart can easily cope with altered circulatory demand, e.g. during increase in blood pressure, exercise, etc. [26]. Low overall HRV is linked to poor cardiovascular prognosis indicating that the heart rhythm is loosely controlled and highly dependent on the local intrinsic pacemaker cells firing in mostly random patterns. Similarly to the changes in the overall HRV, represented by SDNN and TSP, the both indexes of parasympathetic activity: rMSSD (Fig 4D) and HF (Fig 5D) were significantly increased by allopurinol in hypoxia and in minor extent in hyperoxia. According to the Porges' hypothesis [46] confirmed by Carnevali et al.[47] prominent cardiac vagal discharge is indicative of high autonomic flexibility and the capability of the parasympathetic nervous system to generate adequate responses to environmental challenges by modifying HR, respiration and arousals. High level of vagal modulation that can effectively counteract sympathetic drive and reduces vulnerability to ventricular arrhythmias and sudden cardiac death [26,39,40].

XO inhibition did not significantly inflict a sympathovagal balance in hypoxia and hyperoxia as shown by unchanged HRV indexes: LF/HF (Fig 5B) and SDNN/rMSSD (Fig 4B). However compared to hypoxia allopurinol reduced the relative sympathetic contribution in autonomic control in the following hyperoxia as shown by significantly decreased LF/HF and a decreasing tendency of SDNN/rMSSD. Since predominance of sympathetic control is associated with an increased risk of severe cardiovascular events [24], its suppression is potentially beneficial. Our protocol of hypoxia followed by hyperoxia does not imply severe organ injury in ischemic or oxidative stress conditions but to some extent it may be related to a non-critical myocardial ischemia/reperfusion. It should be mentioned that XO inhibition was recently reported to protect against cardiac diastolic dysfunction evoked by ischemia, which was accompanied by normalization of previously prolonged ventricular repolarization [48]. Therefore, our data supports the recommendations for allopurinol use in prevention of ischemia/reperfusion-driven injury [49,50].

Hypoxic challenge resulted in a significantly decreased HR, and an increase of corresponding mean RRi (Fig 4A). The response was maintained during following hyperoxia and 1-hour normoxic recovery. A decrease of HR induced by hypoxia represents a primary adaptive response phylogenetically well conserved, that is commonly displayed by rats. A decreasing trend of LF/HF and SDNN/rMSSD ratios (Figs 4B and 5B) suggests that hypoxia may promote vagal domination. Observed profile of the HRV-response to chemoreflex activation with predominant vagal component resembles mammalian diving reflex which initiates during breath holding.

The regulatory effects of XO inhibition looks ambiguous. A further increase in RRi suggesting increased vagal dominance (Fig 4A) over sinoatrial pacemaker, was associated with a modest increase of LF/HF and SDNN/rMSSD ratios, that in contrast is indicative for sympathetic gain. Possible explanation of this inconsistency is that in case of marked simultaneous activation of sympathetic and vagal efferents, which is plausible looking at our HRV data (a significant increase of both LF and HF) the sympathetic impact on HR is negligible. Such interpretation is in agreement with a recent report showing that hypoxic challenges in conscious rats did not cause relevant change in lumbar nerve activity (SNA) [51]. Earlier reports were inconsistent on the HR-response to hypoxia in rats: an increase [52,53], no change [52] or a decrease of HR were documented [36,54]. It should be emphasized that HR-responses to hypoxia in rats were mostly reported from experiments carried out during post-surgical stress, in anesthesia or in animals with a relatively high basal sympathetic tone [47,55,56]. In small rodents such as rats or mice, the sympathovagal balance is highly vulnerable to any extrinsic or intrinsic challenges and is easily shifted towards sympathetic predominance with reduced vagal tone [57,58]. In such conditions hypoxia commonly results in an increase of HR. The regulatory meaning of HR is not obvious in rats. HR that inversely correlates with overall HRV represented by SDNN or TSP, is generally accepted as a simple index of the sympathovagal balance. On the other hand, it is estimated that HR accounts for less than 30% of the HRV [56] and in experimental rodent models this relationship is even much weaker [59]. Based on our more than 30-year experimental experience with 10- to 20-week-old Wistar rats, we have pointed out that unrestrained quasi-stationary conditions are prerequisite for acquisition of reliable data related to cardiovascular regulation [19,22]. Post-surgical stress, anxiety, new environmental conditions and related curiosity, or general anesthesia profoundly disturbs and usually suppress autonomic discharge and regulatory responses. We previously concluded that basal HR above 400 beats/min was indicative of pathology or excessive stress, hence unacceptable for *in vivo* studies related to neurogenic cardiovascular regulation [60]. Therefore our experiments were performed on thoroughly acclimatized animals in possibly unrestrained stationary conditions. It should be stressed that in the current set of experiments the baseline HR did not exceed 370 beats/min in any rat and the mean HR was by 50 to 150 beats/min slower compared to majority reports referring to the rat studies. So we are convinced that we dealt with really unrestrained rats what is crucial for a relevant evaluation of autonomic regulation from HRV.

Suppression of autonomic discharge, resulting in a reduced regulatory control over sinus node pacemaker or a relative increase of the sympathetic component, are well established and sensitive risk factors of severe cardiac dysrhythmias and sudden death [24–26]. Theoretically, suppression of ROS by an antioxidant, e.g. allopurinol, may result in chemoreflex activation in a way corresponding to conditions of oxygen deficiency with activation of the sympathetic component of the autonomic regulation of the heart [61]. This was, however, not the case in this study, probably due to different ROS produced by XO from those prevalently sensed by carotid body chemoreceptors. In the ROS-based model of oxygen sensing, NADPH oxidase-derived superoxide was suggested as a major signaling molecule [16], while the H_2_O_2_ is a major ROS product of XO [62]. Lack of evident regulatory effect of XO inhibition in the resting normoxia corresponds to the only previous human study report based on a non-uniform group of patients with congestive heart failure chronically supplemented with allopurinol[63]. Since enhanced XO activity in oxygen deficiency conditions promotes oxidative stress related to increased risk of severe cardiac events, observed modulatory effect of allopurinol in hypoxia may prove to be of particular importance—with improved autonomic control upon the heart including increased vagal activity. Thus our study supports earlier suggestions that lowering uric acid might be a promising therapeutic strategy to reduce sympathetic cardiovascular predominance observed in ischemia [64], heart failure [63] and other conditions of autonomic dysfunction [65].

As shown in Fig 1 allopurinol effectively inhibited XO activity. The lowest XO activity in the control group was almost four times higher compared to rats supplemented with allopurinol. Significantly lower isoprostane 8-iso-PGF2α serum concentration (Fig 3A) in rats pretreated with allopurinol indicates that XO inhibition reduced oxidative stress. The causal relationship between XO inhibition and resulting protection from lipid peroxidation was strengthened by a significant inverse significant correlation between residual XO activity in rats supplemented with allopurinol and 8-iso-PGF2α concentration. XO inhibition had no effect on protein carbonyl group (Fig 3B) suggesting that plasma proteins were not the major target of XO-derived ROS. On the other hand it has already been shown in the rat models of isoproterenol-driven oxidative stress resulting in myocardial damage [66] or consequences of oxidative stress in diabetes [67] that allopurinol-driven effects may simultaneously affect the structure and subsequently function of lipids as well as proteins–in plasma and the heart alike. Observed changes include prevention of lipid peroxidation and protein oxidation in addition to normalization of pathological increase of catalase and GPx in challenged rats. As the study was primarily focused on neurogenic regulatory mechanisms, but not the intrinsic activity of the heart pacemakers which were in fact the effectors of that regulation, we restricted our assays related to oxidative stress to plasma. Interestingly XO inhibition was related to an increase of enzymatic antioxidant defense after three-day hypoxic/hyperoxic challenge (Fig 2). The observed trend of increased activity of all three assayed enzymes: SOD, CAT and GPx in rats injected with allopurinol correspond to the recent study referred to the effects of allopurinol in rats challenged with ischemic reperfusion injury [68]. SOD, CAT and GPx are the crucial enzymes of the cell antioxidant defense system. Although the mammalian antioxidant enzymes are mostly constitutive [69] their expression was also reported to be partially controlled by ROS such as O_2_^•−^, H_2_O_2_ or other peroxides [70]. Accordingly, XO inhibition should suppress enzymatic antioxidant defense. On the other hand ROS were documented to inactivate the enzymatic antioxidants. SOD was found to be effectively inactivated by H_2_O_2_, a major ROS product of XO [71], CAT by O_2_^•−^ and OH^−^[71–73], and GPx mainly by OH^−^[71]. In light of our data it seems that protective effects of XO inhibition prevailed and provided additional line of antioxidant defense.

## Limitations of the study

The major issue of the experimental protocol was to provide experimental conditions for reliable assessment of undisturbed autonomic regulation with HRV analysis. In particular we tried to avoid excessive stress, injury or uncontrolled inflammation. For that reason the only invasive intervention was subcutaneous implantation of ECG electrodes of possibly minimal size. Such physiological variables of the regulatory relevance as arterial pressure or respiration has not been assessed due to high risk of unacceptable invasiveness of the surgical procedures. Blood samples useful for biochemical analyses were not taken during the experimental procedure. As a consequence our data are exclusively based on the ECG recording during the protocol and the biochemical assays from the final point of the experiment. Extrapolation of the obtained here data to humans needs caution. In the rat basal XO activity is much higher compared to humans and its devastating role as an oxidant is much higher [74]. However, data from human studies showed that during ischemia or postischemic reperfusion generation of ROS by XO is rapidly increased so that the enzyme is an important trigger of oxidative stress.

## Summary

Concluding, allopurinol reduced oxidative stress evoked by hypoxia and post-hypoxic hyperoxia in conscious unrestrained rats. In standard normoxic conditions XO inhibition does not seem to interfere with neurogenic heart regulation. Thus the concept that allopurinol acting as an oxidant perturbing oxygen sensing to activate chemoreflex has not been supported by our data. Moreover, in hypoxia, when chemoreflex is activated, XO inhibitor, allopurinol enhanced autonomic influence on the heart rhythm with increased vagal role. The observed regulatory effects provide an argument for using XO inhibitors in hypoxic conditions.

## Supporting information

S1 Appendix Database of HRV for allopurinol and control group. (XLSX)

## References

[1] Rahman K. Studies on free radicals, antioxidants, and co-factors. Clin Interv Aging. 2007;2(2):219–36. 18044138 PMC2684512

[2] Stone PH. Allopurinol: A new anti-ischemic role for an old drug. J Am Coll Cardiol. 2011;58(8):829–30. doi: 10.1016/j.jacc.2011.02.072 21835318 10.1016/j.jacc.2011.02.072

[3] Pacher P, Nivorozhkin A, Szabo C. Therapeutic Effects of Xanthine Oxidase Inhibitors: Hum Physiol. 2006;58(1):87–114. 10.1124/pr.58.1.6 PMC2233605 16507884

[4] Farquharson CAJ, Butler R, Hill A, Belch JJF, Struthers AD. Allopurinol improves endothelial dysfunction in chronic heart failure. Circulation. 2002;106(2):221–6. 12105162 10.1161/01.cir.0000022140.61460.1d

[5] Berry CE, Hare JM. Xanthine oxidoreductase and cardiovascular disease: molecular mechanisms and pathophysiological implications. J Physiol. 2004;555(Pt 3):589–606. doi: 10.1113/jphysiol.2003.055913 14694147 10.1113/jphysiol.2003.055913 PMC1664875

[6] Talwar S, Sandeep JA, Choudhary SK, Velayoudham D, Lakshmy R, Kasthuri JM, et al Effect of preoperative administration of allopurinol in patients undergoing surgery for valvular heart diseases. Eur J Cardiothorac Surg 2010;38:86–90 doi: 10.1016/j.ejcts.2010.01.027 20188583 10.1016/j.ejcts.2010.01.027

[7] Singh JA, Cleveland J. Allopurinol and the risk of ventricular arrhythmias in the elderly: a study using US Medicare data. BMC Med. 2017;15(1):59 doi: 10.1186/s12916-017-0816-6 28327188 10.1186/s12916-017-0816-6 PMC5361697

[8] Crome R, Manning AS. Experimental Studies Allopurinol and Reperfusion-Induced Arrhythmias: Increased Protection By Simultaneous Administration of Anti-Oxidant Enzymes. Cardiovasc Drugs Ther. 1988;2:295–304. 3154913 10.1007/BF00054636

[9] Bjelakovic G, Nikolova D. D, Gluud LL, Simonetti RG, Gluud C. Mortality in randomized trials of antioxidant supplements for primary and secondary prevention: systematic review and meta-analysis. JAMA. 2007;297(8):842–57. doi: 10.1001/jama.297.8.842 17327526 10.1001/jama.297.8.842

[10] Myung SK, Ju W, Cho B, Oh SW, Park SM, Koo BK, et al Efficacy of vitamin and antioxidant supplements in prevention of cardiovascular disease: systematic review and meta-analysis of randomised controlled trials. Bmj. 2013;346:12 10.1136/bmj.f10 PMC3548618 23335472

[11] Klein EA, Thompson IM, Tangen CM, Crowley JJ, Lucia MS, Goodman PJ, et al Vitamin E and the Risk of Prostate Cancer. J Am Med Assoc. 2011 10 12; 306(14):1549–56. 10.1001/jama.2011.1437 PMC4169010 21990298

[12] Agapito MT, Sanz-Alfayate G, Gomez-Niño A, Gonzalez C, Obeso A. General redox environment and carotid body chemoreceptor function. Am J Physiol Cell Physiol. 2009;296(3):C620–31. doi: 10.1152/ajpcell.00542.2008 19144860 10.1152/ajpcell.00542.2008

[13] Nunes FC, Ribeiro TP, França-Silva MS, Medeiros IA, Braga VA. Superoxide scavenging in the rostral ventrolateral medulla blunts the pressor response to peripheral chemoreflex activation. Brain Res. 2010;1351:141–9. doi: 10.1016/j.brainres.2010.07.001 20621069 10.1016/j.brainres.2010.07.001

[14] Iturriaga R, Del Rio R, Idiaquez J, Somers VK. Carotid body chemoreceptors, sympathetic neural activation, and cardiometabolic disease. Biol Res. 2016; 49:13 doi: 10.1186/s40659-016-0073-8 26920146 10.1186/s40659-016-0073-8 PMC4768417

[15] Ortiz FC, Del Rio R, Ebensperger G, Reyes VR, Alcayaga J, Varas R, et al Inhibition of rat carotid body glomus cells TASK-like channels by acute hypoxia is enhanced by chronic intermittent hypoxia. Respir Physiol Neurobiol. 2013;185(3):600–7. doi: 10.1016/j.resp.2012.11.015 23219812 10.1016/j.resp.2012.11.015

[16] Li YL, Gao L, Zucker IH, Schultz HD. NADPH oxidase-derived superoxide anion mediates angiotensin II-enhanced carotid body chemoreceptor sensitivity in heart failure rabbits. Cardiovasc Res. 2007;75(3):546–54. doi: 10.1016/j.cardiores.2007.04.006 17499230 10.1016/j.cardiores.2007.04.006 PMC2062532

[17] Prabhakar NR. Sensing hypoxia: physiology, genetics and epigenetics. J Physiol. 2013;591(9):2245–57. doi: 10.1113/jphysiol.2012.247759 23459758 10.1113/jphysiol.2012.247759 PMC3650692

[18] Del Rio R. The carotid body and its relevance in pathophysiology. Exp Physiol. 2015;100(2):121–3. http://doi.wiley.com/10.1113/expphysiol.2014.079350 25767839 10.1113/expphysiol.2014.079350

[19] Gawiński Ł, Wierzba TH. Wpływ histydyny na zmienność rytmu serca u szczura. Ann Acad Medicae Gedanensis. 2006;36:53–61.

[20] Wierzba TH, Borkowski T, Gawiński Ł. Do antioxidants play with heart rate variability? Proc Prog Biomed Sci. 2004;1:11–8.

[21] Borkowski T, Wierzba TH. Effect of ascorbic acid on heart rate variability (HRV) in rats as an example of the feasible regulatory potential of antioxidants. Pol J Thorac Cardiovasc Surg. 2004;1(4):191–8.

[22] Wierzba TH. Effect of free-radical suppression on cardiovascular regulation and endurance capacity in hypertensive rats. Ann Acad Medicae Gedanensis. 2007;34(suppl. 4).

[23] Hare JM, Mangal B, Brown J, Fisher C, Freudenberger R, Colucci WS, et al Impact of Oxypurinol in Patients With Symptomatic Heart Failure. Results of the OPT-CHF Study. J Am Coll Cardiol. 2008;51(24):2301–9. doi: 10.1016/j.jacc.2008.01.068 18549913 10.1016/j.jacc.2008.01.068

[24] Guidelines Heart rate variability Standards of measurement, physiological interpretation, and clinical use. Eur Heart J. 1996;17:354–81. 8737210

[25] Malpas SC. Neural influences on cardiovascular variability: possibilities and pitfalls. Am J Physiol Heart Circ Physiol. 2002;282(1):H6–20. doi: 10.1152/ajpheart.2002.282.1.H6 11748042 10.1152/ajpheart.2002.282.1.H6

[26] Parati G, Faini A VM. Blood pressure variabiliy: Its measurement and significance in hypertension. Curr Hypertens Rep. 2006;8:199–204. 17147917 10.1007/s11906-006-0051-6

[27] Reyes del Paso GA, Langewitz W, Mulder LJM, van Roon A, Duschek S. The utility of low frequency heart rate variability as an index of sympathetic cardiac tone: A review with emphasis on a reanalysis of previous studies. Psychophysiology. 2013;50: 477–87. doi: 10.1111/psyp.12027 23445494 10.1111/psyp.12027

[28] Stein PK, Domitrovich PP, Hui N, Rautaharju P, Gottdiener J. Sometimes higher heart rate variability is not better heart rate variability: Results of graphical and nonlinear analyses. J Cardiovasc Electrophysiol. 2005;16(9):954–9. doi: 10.1111/j.1540-8167.2005.40788.x 16174015 10.1111/j.1540-8167.2005.40788.x

[29] Wang HM, Huang SC. SDNN/RMSSD as a surrogate for LF/HF: A revised investigation. Model Simul Eng. 2012;2012:1–8.

[30] Szasz T, Linder AE, Davis RP, Burnett R, Fink GD, Watts SW. Allopurinol does not Decrease Blood Pressure or Prevent the Development of Hypertension in the Doca-Salt Rat Model. J Cardiovasc Pharmacol. 2010;56(6):627–34. doi: 10.1097/FJC.0b013e3181f80194 20881613 10.1097/FJC.0b013e3181f80194 PMC3049193

[31] Yamamoto Y, Ogino K, Igawa G, Matsuura T, Kaetsu Y, Sugihara S, et al Allopurinol reduces neointimal hyperplasia in the carotid artery ligation model in spontaneously hypertensive rats. Hypertens Res. 2006;29(11):915–21. doi: 10.1291/hypres.29.915 17345792 10.1291/hypres.29.915

[32] Gürbüz Özgür B, Aksu H, Birincioʇlu M, Dost T. Antidepressant-like effects of the xanthine oxidase enzyme inhibitor allopurinol in rats. A comparison with fluoxetine. Pharmacol Biochem Behav. 2015;138:91–5. doi: 10.1016/j.pbb.2015.09.016 26409178 10.1016/j.pbb.2015.09.016

[33] Day RO, Graham GG, Hicks M, McLachlan AJ, Stocker SL, Williams KM. Clinical pharmacokinetics and pharmacodynamics of allopurinol and oxypurinol. Clin Pharmacokinet. 2007;46(8):623–44. doi: 10.2165/00003088-200746080-00001 17655371 10.2165/00003088-200746080-00001

[34] Oliver CN. Inactivation of enzymes and oxidative modification of proteins by stimulated neutrophils. Arch Biochem Biophys. 1987;253(1):62–72. 2880566 10.1016/0003-9861(87)90637-0

[35] Aebi H. [13] Catalase in vitro. Methods Enzymol. 1984;105(C):121–6. 6727660 10.1016/s0076-6879(84)05016-3

[36] Zajączkowski S, Smolińska M, Badtke P, Wierzba TH. Time-Domain and Spectral Analysis of Heart Rate Variability in Rats Challenged with Hypoxia. Comput Cardiol (2010). 2014;41:785–8.

[37] Carnevali L, Trombini M, Porta A, Montano N, de Boer SF, Sgoifo A. Vagal Withdrawal and Susceptibility to Cardiac Arrhythmias in Rats with High Trait Aggressiveness. PLoS One. 2013;8(7):1–11. 10.1371/journal.pone.0068316 PMC3701673 23861886

[38] Buntrock RE. The Merck Index: An Encyclopedia of Chemicals, Drugs, and Biologicals, Fourteenth Edition Edited by Maryadele J. O’Neil, Patricia E. Heckelman, Cherie B. Koch, and Kristin J. Roman. Merck & Co., Inc: Whitehouse Station, New Jersey, 2006. American Chemical Society; 2007

[39] Volders PGA. Novel insights into the role of the sympathetic nervous system in cardiac arrhythmogenesis. Hear Rhythm. 2010;7(12):1900–6. 10.1016/j.hrthm.2010.06.003 20570754

[40] Carnevali L, Trombini M, Graiani G, Madeddu D, Quaini F, Landgraf R, et al Low vagally-mediated heart rate variability and increased susceptibility to ventricular arrhythmias in rats bred for high anxiety. Physiol Behav. 2014;128:16–25. doi: 10.1016/j.physbeh.2014.01.033 24518868 10.1016/j.physbeh.2014.01.033

[41] Altimiras J. Understanding autonomic sympathovagal balance from short-term heart rate variations. Are we analyzing noise? Vol. 124, Comparative Biochemistry and Physiology—A Molecular and Integrative Physiology. 1999 p. 447–60. 10.1016/s1095-6433(99)00137-3 10682243

[42] Zigon-Japundzic N, Jovanovic A. Blood Pressure And Heart Rate Spectral Changes Induced By The Modulation Of The Cholinergic Transmission By Physostigmine And Neostigmine. Jugoslav Physiol Pharmacol Acta. 1998;34(1):111–20.

[43] Baker JE, Felix CC, Olinger GN, Kalyanaraman B. Myocardial ischemia and reperfusion: direct evidence for free radical generation by electron spin resonance spectroscopy. Proc Natl Acad Sci USA. 1988;85(8):2786–9. 2833754 10.1073/pnas.85.8.2786 PMC280084

[44] Zweier JL, Talukder MAH. The role of oxidants and free radicals in reperfusion injury. Vol. 70, Cardiovascular Research. 2006 p. 181–90. doi: 10.1016/j.cardiores.2006.02.025 16580655 10.1016/j.cardiores.2006.02.025

[45] Schultz HD, Li YL. Carotid body function in heart failure. Respir Physiol Neurobiol. 2007;157(1):171–85. doi: 10.1016/j.resp.2007.02.011 17374517 10.1016/j.resp.2007.02.011 PMC1965591

[46] Porges SW. Porges, 1995, Cardiac Vagal Tone A physiological index of stress. Vol. 19, Neuroscience and Biobehavioral Reviews. 1995 p. 225–33. 7630578 10.1016/0149-7634(94)00066-a

[47] Carnevali L, Sgoifo A. Vagal modulation of resting heart rate in rats: The role of stress, psychosocial factors, and physical exercise. Front Physiol. 2014;5 MAR(3):1–12. 24715877 10.3389/fphys.2014.00118 PMC3970013

[48] El-Bassossy HM, Shaltout HA. Allopurinol alleviates hypertension and proteinuria in high fructose, high salt and high fat induced model of metabolic syndrome. Transl Res. 2015;165(5):621–30. doi: 10.1016/j.trsl.2014.11.008 25528722 10.1016/j.trsl.2014.11.008

[49] Angdin M, Settergren G, Starkopf J, Zilmer M, Zilmer K, Vaage J. Protective effect of antioxidants on pulmonary endothelial function after cardiopulmonary bypass. J Cardiothorac Vasc Anesth. 2003;17(3):314–20. 12827578 10.1016/s1053-0770(03)00053-3

[50] Lee BE, Toledo AH, Anaya-Prado R, Roach RR, Toledo-Pereyra LH. Allopurinol, xanthine oxidase, and cardiac ischemia. J Investig Med. 2009;57(8):902–9. doi: 10.2310/JIM.0b013e3181bca50c 19794315 10.2310/JIM.0b013e3181bca50c

[51] Martin Muntzel, Jaclquelin Loera, Gregory Coffee, Stephen J Lewis, Paulina Getsy A. Intermittent Hypoxic Challenge in Conscious Rats Elevates Heart Rate But Does Not Increase Lumbar Sympathetic Nerve Activity. FASEB J. 2017 4 1;31(1 Supplement):841.13–841.13.

[52] Murasato Y, Hirakawa H, Harada Y, Nakamura T, Hayashida Y. Effects of systemic hypoxia on R-R interval and blood pressure variabilities in conscious rats. Am J Physiol. 1998;275(3 Pt 2):H797–804. 9724282 10.1152/ajpheart.1998.275.3.H797

[53] S Sugimura M, Hirose Y, Hanamoto H, Okada K, Boku A, Morimoto Y, et al Influence of acute progressive hypoxia on cardiovascular variability in conscious spontaneously hypertensive rats. Auton Neurosci Basic Clin. 2008;141(1–2):94–103. 10.1016/j.autneu.2008.05.008 PMC2941824 18599365

[54] Gonçalves H, Henriques-Coelho T, Bernardes J, Rocha AP, Nogueira A, Leite-Moreira A. Linear and nonlinear heart-rate analysis in a rat model of acute anoxia. Physiol Meas. 2008;29(9):1133–43. doi: 10.1088/0967-3334/29/9/010 18784391 10.1088/0967-3334/29/9/010

[55] Matchett G, Wood P. General anesthesia suppresses normal heart rate variability in humans. Chaos. 2014;24(2):23129 http://dx.doi.org/10.1063/1.4882395 10.1063/1.4882395 24985443

[56] Billman GE. The LF/HF ratio does not accurately measure cardiac sympatho-vagal balance. Vol. 4, Front Physiology. 2013;4(26). 10.3389/fphys.2013.00026 PMC3576706 23431279

[57] Mansier P, Clairambault J, Charlotte N, Médigue C, Vermeiren C, LePape G, et al Linear and non-linear analysis of heart rate variability: a minireview. Vol. 31, Cardiovasc Res. 1996 p. 371–9. 8681324

[58] Swynghedauw B, Jasson S, Chevalier B, Clairambault J, Hardouin S, Heymes C, et al Heart rate and heart rate variability, a pharmacological target. Cardiovasc Drugs Ther. 1997;10(6):677–85. 9110110 10.1007/BF00053024

[59] Sacha J, Pluta W. Different methods of heart rate variability analysis reveal different correlations of heart rate variability spectrum with average heart rate. J Electrocardiol. 2005;38(1):47–53. doi: 10.1016/j.jelectrocard.2004.09.015 15660347 10.1016/j.jelectrocard.2004.09.015

[60] Labudda O, Wierzba T, Sobolewski D, Kowalczyk W, Śleszyńska M, Gawínski Ł, et al New bradykinin analogues substituted in positions 7 and 8 with sterically restricted 1-aminocyclopentane-1-carboxylic acid. J Pept Sci. 2006;12(12):775–9. doi: 10.1002/psc.812 17131289 10.1002/psc.812

[61] Gonzalez C, Sanz-Alfayate G, Agapito MT, Gomez-Niño A, Rocher A, Obeso A. Significance of ROS in oxygen sensing in cell systems with sensitivity to physiological hypoxia. Respir Physiol Neurobiol. 2002;132:17–41. 12126693 10.1016/s1569-9048(02)00047-2

[62] Kelley EE, Khoo NKH, Hundley NJ, Malik UZ, Freeman BA, Tarpey MM. Hydrogen peroxide is the major oxidant product of xanthine oxidase. Free Radic Biol Med. 2010;48(4):493–8. doi: 10.1016/j.freeradbiomed.2009.11.012 19941951 10.1016/j.freeradbiomed.2009.11.012 PMC2848256

[63] Shehab AMA, Butler R, MacFadyen RJ, Struthers AD, et al A placebo-controlled study examining the effect of allopurinol on heart rate variability and dysrhythmia counts in chronic heart failure. Br J Clin Pharmacol. 2001;51(4):329–34. doi: 10.1046/j.1365-2125.2001.01361.x 11318768 10.1046/j.1365-2125.2001.01361.x PMC2014457

[64] Chouchou F, Pichot V, Barthélémy J-C, Bastuji H, Roche F. Cardiac Sympathetic Modulation in Response to Apneas/Hypopneas through Heart Rate Variability Analysis. PLoS One. 2014;9(1):e86434 doi: 10.1371/journal.pone.0086434 24466093 10.1371/journal.pone.0086434 PMC3899280

[65] Hatayama M, Sumida C, Kurajoh M, Shiraishi J, Okazaki H, Shoji T, et al Acute Effects of Oral Tofisopam on Plasma Concentration and Urinary Excretion of Uric Acid and Oxypurinol "Preliminary Communication". Curr Clin Pharmacol. 2015;10(2):160–4. doi: 10.2174/1574884710666150126143421 25619490 10.2174/1574884710666150126143421

[66] Sagor MAT, Tabassum N, Potol MA, Alam MA. Xanthine oxidase inhibitor, allopurinol, prevented oxidative stress, fibrosis, and myocardial damage in isoproterenol induced aged rats. Oxid Med Cell Longev. 2015. 10.1155/2015/478039 PMC4475550 26137187

[67] Desco M-C, Asensi M, Má Rquez R, Martínez-Valls J, Ximo Vento M, Pallardó F V, et al Xanthine Oxidase Is Involved in Free Radical Production in Type 1 Diabetes Protection by Allopurinol. Diabetes. 2002;51(4):1118–24. 11916934 10.2337/diabetes.51.4.1118

[68] Ansari MA, Hussain SK, Mudagal MP, Goli D. Neuroprotective effect of allopurinol and nimesulide against cerebral ischemic reperfusion injury in diabetic rats. Eur Rev Med Pharmacol Sci. 2013;17(2):170–8. 23377804

[69] Desaint S, Luriau S, Aude JC, Rousselet G, Toledano MB. Mammalian antioxidant defenses are not inducible by H2O2. J Biol Chem. 2004;279(30):31157–63. doi: 10.1074/jbc.M401888200 15155764 10.1074/jbc.M401888200

[70] Franco AA, Odom RS, Rando TA. Regulation of antioxidant enzyme gene expression in response to oxidative stress and during differentiation of mouse skeletal muscle. Free Radic Biol Med. 1999;27(9–10):1122–32. 10569645 10.1016/s0891-5849(99)00166-5

[71] Pigeolet E, Corbisier P, Houbion A, Lambert D, Michiels C, Raes M, et al Glutathione peroxidase, superoxide dismutase, and catalase inactivation by peroxides and oxygen derived free radicals. Mech Ageing Dev. 1990;51(3):283–97. 2308398 10.1016/0047-6374(90)90078-t

[72] Kono Y, Fridovich I. Superoxide radical inhibits catalase. J Biol Chem. 1982;257(10):5751–4. 6279612

[73] Bray RC, Cockle SA, Fielden EM, Roberts PB, Rotilio G, Calabrese L. Reduction and inactivation of superoxide dismutase by hydrogen peroxide. Biochem J 1974;139(1):43–8. 4377099 10.1042/bj1390043 PMC1166249

[74] Muxfeldt M, Schaper W. The activity of xanthine oxidase in heart of pigs, guinea pigs, rabbits, rats, and humans. Basic Res Cardiol. 1987;82(5):486–92. 3426527 10.1007/BF01907096

